# Generation of a BAC-based physical map of the melon genome

**DOI:** 10.1186/1471-2164-11-339

**Published:** 2010-05-28

**Authors:** Víctor M González, Jordi Garcia-Mas, Pere Arús, Pere Puigdomènech

**Affiliations:** 1 Molecular Genetics Department, Center for Research in Agricultural Genomics CRAG (CSIC-IRTA-UAB), Jordi Girona, 18-26, 08034 Barcelona, Spain; 2 Plant Genetics Department, IRTA, Center for Research in Agricultural Genomics CRAG (CSIC-IRTA-UAB), Carretera de Cabrils Km 2, 08348 Barcelona, Spain

## Abstract

**Background:**

*Cucumis melo *(melon) belongs to the Cucurbitaceae family, whose economic importance among horticulture crops is second only to Solanaceae. Melon has high intra-specific genetic variation, morphologic diversity and a small genome size (450 Mb), which make this species suitable for a great variety of molecular and genetic studies that can lead to the development of tools for breeding varieties of the species. A number of genetic and genomic resources have already been developed, such as several genetic maps and BAC genomic libraries. These tools are essential for the construction of a physical map, a valuable resource for map-based cloning, comparative genomics and assembly of whole genome sequencing data. However, no physical map of any Cucurbitaceae has yet been developed. A project has recently been started to sequence the complete melon genome following a whole-genome shotgun strategy, which makes use of massive sequencing data. A BAC-based melon physical map will be a useful tool to help assemble and refine the draft genome data that is being produced.

**Results:**

A melon physical map was constructed using a 5.7 × BAC library and a genetic map previously developed in our laboratories. High-information-content fingerprinting (HICF) was carried out on 23,040 BAC clones, digesting with five restriction enzymes and SNaPshot labeling, followed by contig assembly with FPC software. The physical map has 1,355 contigs and 441 singletons, with an estimated physical length of 407 Mb (0.9 × coverage of the genome) and the longest contig being 3.2 Mb. The anchoring of 845 BAC clones to 178 genetic markers (100 RFLPs, 76 SNPs and 2 SSRs) also allowed the genetic positioning of 183 physical map contigs/singletons, representing 55 Mb (12%) of the melon genome, to individual chromosomal loci. The melon FPC database is available for download at http://melonomics.upv.es/static/files/public/physical_map/.

**Conclusions:**

Here we report the construction of the first physical map of a Cucurbitaceae species described so far. The physical map was integrated with the genetic map so that a number of physical contigs, representing 12% of the melon genome, could be anchored to known genetic positions. The data presented is already helping to improve the quality of the melon genomic sequence available as a result of a project currently being carried out in Spain, adopting a whole genome shotgun approach based on 454 sequencing data.

## Background

*Cucumis melo *(melon) is an important crop worldwide. It belongs to the Cucurbitaceae family, which also includes cucumber, watermelon, pumpkin and squash, and whose economic importance, among horticulture crops, is second only to Solanaceae. Melon has 2n = 24 chromosomes and its haploid genome contains 4.5 × 10^8 ^bp, only three times larger than the *Arabidopsis *genome and similar in size to the rice genome [1]. Melon has high intra-specific genetic variation and morphologic diversity [2,3] that, together with its small genome size, make it suitable for a great variety of molecular and genetic studies that can lead to the development of tools for crop improvement. Genomics approaches to melon breeding have already been successfully applied to the molecular characterization of important agronomic traits such as pathogen resistances [4-6]. Recent research has increased the genetic and genomic resources for melon [7], such as the sequencing of ESTs [8,9], the construction of BAC libraries [10,11], the development of an oligo-based microarray [12], the production of melon mutation libraries for TILLING analyses [13,14] or the development of a collection of near isogenic lines (NILs) [15]. Several genetic maps have also been reported for melon and a consensus genetic map, obtained by merging available maps using a common set of SSRs as anchor markers, has recently been obtained by the International Cucurbit Genomics Initiative (ICuGI) [16-20,9]. The MELONOMICS project, aimed at the sequencing of the complete melon genome following a whole-genome shotgun strategy that makes use of 454 sequencing data, has recently been started in Spain.

A double-haploid line (DHL) population from the cross between the Korean accession PI 161375 and the *inodorus *type 'Piel de sapo' T111 was the basis for the construction of 1) a BAC library with an average insert size of 139 kb, representing 5.7 genome equivalents of the *C. melo *haploid genome and 2) a genetic map with around 700 markers, of which more than 500 are gene-based markers (SNP, RFLP and SSR) [10,19,20]. A fraction of the genetic markers in the melon genetic map has been mapped at low resolution using the bin-mapping strategy [19]. These tools are essential for the construction of a melon physical map, a resource that greatly facilitates assembly and refinement of whole genome sequencing data and that can also be used to define a minimum tilling path of BAC clones in BAC-to-BAC genome sequencing strategies. The utility of physical maps has been reported by several classical genome sequencing projects such as those of human [21], *Arabidopsis *[22] and rice [23]. These first physical maps were constructed using restriction enzyme digestion of BAC DNA and agarose gel electrophoresis, with the restriction patterns analyzed by FingerPrinted Contigs (FPC) software to obtain contigs of BAC clones [24,25]. As an alternative to agarose gel-based fingerprinting methods, which are time-consuming and can often lead to poor maps due to the need for manual band calling and the comparatively low resolution of agarose gels, fluorescence-labeled capillary electrophoresis methods have been developed to produce larger, more accurate contigs using larger sets of BAC clones [26-28]. The evaluation of five different fingerprinting methods lead to the conclusion that the high-information-content fingerprinting method (HICF) with five-enzyme digestion plus SNaPshot labeling is the most effective [26,29]. HICF methods have already been applied to the development of physical maps of several species such as catfish, apple, grape, wheat, *B. rapa*, peach, papaya, trout, *Brachypodium *or maize but no physical maps have as yet been developed for any Cucurbitaceae species [30-39].

However, for physical maps to be useful, BAC contigs need to be anchored to genetic maps in order to establish the relative genomic position of the maximum number of physical map fragments. The anchored contigs can then be used as seed points for bidirectional chromosome sequencing, a crucial resource to fill gaps and refine assemblies of genome draft sequencing data. PCR-based methods combined with adequate pooling of BAC DNA samples have proved to be very cost- and time-effective for the anchoring of BAC libraries to genetic maps [40-43].

Here we report the construction of the first physical map of a Cucurbitaceae species described so far, using a 5.7× BAC library and a genetic map previously developed in our laboratories. HICF was carried out on 23,040 BAC clones, digesting with five restriction enzymes and SNaPshot labeling, followed by contig assembly with FPC software. Anchoring the BAC library to the genetic map has also allowed the genetic positioning of 183 physical contigs/singletons to chromosomal loci. The melon physical map FPC database is available for download at http://melonomics.upv.es/static/files/public/physical_map/.

## Results and discussion

### BAC fingerprinting and contig assembly

A BAC library from the double-haploid melon line 'PIT92' had been previously constructed in our laboratory with an average insert size of 139 kb and representing 5.7 genome equivalents of the *C. melo *haploid genome (based on an estimated haploid genome size of 445 Mb [1]) [10]. All 23,040 BAC clones, of which 80% are estimated to be non-empty clones, were fingerprinted by digestion with five restriction enzymes and posterior labeling using the SNaPshot Kit. The labeled fragments were sized using an ABI3730 DNA sequencer and the FPB software used to remove background, poor quality fingerprints, vector bands and fingerprints with less than 50 bands in the 50 bp-500 bp range [44]. The resulting 14,484 clones (corresponding to approximately 80% of the clones with insert) had an average of 102 valid bands per fingerprint (see Table 1), which where subsequently subjected to contig assembly using the FPC software with tolerance 0.4 bp [24].

**Table 1:** Summary of the *C. melo *FPC physical map.

Number of clones fingerprinted		23,040
Number of non-empty fingerprinted clones		18,200
Number of clones with successful fingerprints		14,484
Average number of valid bands per clone		102.1
Number of singletons		441
Number of contigs		1,355
**Contig size distribution**		
**100-199**		3
**50-99**		9
**25-49**		102
**10-24**		428
**3-9**		706
**2**		107

**Average contig length (kb)**		300

**Number of contigs with Q clones** **Contigs with ≥ 5% of Q clones**		62 0

**Total physical length of the contigs (Mb)**		407
**Longest contig**		Ctg200 (3.2 Mb)

**Number of genetic markers anchored**		**169**
**Anchored to contigs**		157
**Anchored to singletons**		12

**Number of FPC contigs and singletons linked to** **genetic markers**		**171/18^a^**
**3 markers**		2/0^a^
**2 markers**		11/1^a^
**1 marker**		158/17^a^
**Average length (kb)**		329/101^a^

**Number of markers linked to more than**		
**one FPC contig**		**30**
**Two contigs**		19
**Three contigs**		3
**Four contigs**		1
**1 contig + 1 singleton**		4
**2 contig + 1 singleton**		1
**2 singletons**		2

**Number of contigs/singletons and BACs anchored to chromosomes^b^**	**Contigs/Singletons^c^**	**BACs**
**I**	20 (5.7 Mb)	191
**II**	18 (6.0 Mb)	230
**III**	13 (3.4 Mb)	148
**IV**	18 (4.4 Mb)	176
**V**	9 (2.5 Mb)	203
**VI**	20 (5.9 Mb)	216
**VII**	13 (3.8 Mb)	142
**VIII**	17 (4.9 Mb)	178
**IX**	14 (5.4 Mb)	211
**X**	12 (3.9 Mb)	154
**XI**	16 (5.6 Mb)	228
**XII**	6 (1.2 Mb)	29
**I/IX^d^**	1 (0.3 Mb)	11
**I/XI^d^**	1 (0.5 Mb)	25
**II/VIII^d^**	1 (0.3 Mb)	5
**IV/V^d^**	3 (0.6 Mb)	22
**IV/VI^d^**	1 (0.7 Mb)	46
**TOTAL**	183 (55.0 Mb)	2,215

^a^/: Contigs/singletons

^b ^Contigs or singletons anchored to markers with conflicting genetic positions are not considered

^c ^Length of physical contigs and singletons anchored to each chromosome appears in parenthesis

^d ^Contigs and singletons anchored to markers that map loci in two chromosomes

An initial test to determine the optimal cutoff value, minimizing the contig number while avoiding a large number of questionable clones [Q-clones], was performed with several cutoff values in the 1e-35/1e-65 range. Lower cutoff values mean more stringent assembly conditions that prevent chimeric contig assemblies but at the possible cost of breaking true contigs, increasing the number of contigs and of unassembled clones (singletons). Based on the test results [Table 2], a Sulston score of 1e-45 was chosen for the first automatic assembly. The physical map was built according to a standard iterated procedure with the initial cutoff stringency sufficient to give valid contigs, which are then gradually merged at successively greater cutoff values. Details of the procedure are described in the Methods section and a summary of the successive physical map assemblies is given in Table 2.

**Table 2:** Summary of the *C. melo *physical map FPC assemblies.

Tests for initial cutoff value^a^	Contigs	Singletons	Physical length (Mb)	Q clones^b^							
							
Test 1e-35	1420	1133	398	40/233							
Test 1e-45	1706	1772	421	40/158							
Test 1e-55	1909	2562	432	29/84							
Test 1e-65	2075	3399	438	12/44							
**Assembly** **Steps^c^**	**Contigs**	**Singletons**	**Physical** **length (Mb)**	**Q clones^b^**	**Longest** **contig (Mb)**	**Number of contigs** **(number of contigs with > 5% Q-clones)^d^**					

						**≥ 100**	**99-50**	**49-25**	**24-10**	**9-3**	**= 2**

Initial 1e-45	1706	1772	1.17	421	40/158	0(0)	6(5)	63(36)	393(89)	968(28)	276(0)
DQer (1e-48 to 1e-90)	1777	1923	1.10	428	66/0	0	1	49	412	1027	288
Merge 1e-40	1750	1551	1.10	432	66/0	0	2	51	431	1017	249
Merge 1e-35	1714	1290	1.10	430	66/0	0	2	56	442	998	216
Merge 1e-30	1650	1071	1.4	426	66/0	1	3	62	449	953	182
Merge 1e-25	1577	836	1.5	421	65/0	2	3	69	462	884	157
Merge 1e-20	1491	635	2.8	415	64/0	3	5	79	456	816	132
Merge 1e-15	1355	441	3.2	407	62/0	3	9	102	428	706	107

^a^An initial test to determine the optimal cutoff value was performed with several cutoff values in the 1e-35/1e-65 range

^b^Number of contigs containing ≤/> than 5% of Q-clones

^c^A cutoff value of 1e-45 was used for the initial contig assembly. Consecutive DQer (for contigs containing > 5% Q-clones), end-merge and singleton-merge steps were performed to minimize the number of singletons, contigs and Q-clones

^d^Distribution according to the number of clones belonging to each contig

The final physical map [Table 1] had 1,355 contigs and 441 singletons, with an estimated physical length of 407 Mb (0.9 × coverage of the genome). The longest contig was 3.2 Mb long; the average contig length, 300 kb; 40% of the contigs were made up of the assembly of more than 9 clones; 84% of the contigs contained between 3 and 24 clones, and 62 contigs contained less than the maximum number of Q-clones, 5%. This represents a small percentage of Q-contigs and was the result of the forced breaking of all contigs with more than 5% of Q clones in the first stage of construction. This was achieved by reducing the cutoff to values as low as 1e-99 where necessary. Although this increased the number of contigs and unassembled clones, the reliability of the resulting contigs was greatly improved.

These figures are comparable to those of other plant physical maps recently described. For example, the physical map of *B. rapa*, a species with a genome size of 550 Mb, similar to that of melon, was built by fingerprinting 67,468 BAC clones (×15 genomic equivalents). It has 1,428 contigs of average length 512 kb, 57% of which are made up of more than 9 clones, and the estimated genomic coverage of the map is 1.3 × (725 Mb) [34]. However, the number of singletons (14,001) is more than 30 times higher than that in our map, even though the iterated procedures used to build both maps were similar. The fact that the *B. rapa *library represents 2.6 times more genomic equivalents than the melon could partially explain the differences in singleton number. The analysis of the number of Q clones after each round of construction at different cutoffs suggests that clones remaining as singletons in the *B. rapa *map may not just be due to low quality fingerprints but may come from regions of low coverage in the BAC libraries used [34]. This means that the differences in number of singletons on these physical maps could also be due to differences in the representation of the *B. rapa *and *C. melo *genomes in the different libraries.

As another example, the physical map of papaya, a species with a genome size of 372 Mb, slightly smaller than that of melon, was built by fingerprinting clones representing 13.7 genomic equivalents. It has 963 contigs, 59% of which are made up of more than 9 clones, with an estimated genomic coverage of 0.96 × [36]. The difference in genomic coverage of the libraries could again explain the higher number of singletons, 4,358, ten times higher than in our map.

Regarding the internal structure of the *C. melo *FPC contigs, the comparison of ordered lists of contigs, based on the contig physical length or the contig size (number of clones belonging to the contigs), revealed a high proportion of 'stacked' contigs, that is, contigs containing regions where the depth far exceeds the estimated coverage of the library used (×5.7), possibly due to the non-randomness affecting all libraries constructed by one enzyme restriction of genomic DNA. This poses a problem in that many clones in these stacked contigs do not contribute to extending the physical length of the contig and their fingerprints carry no new information. Also, the visual inspection of contigs revealed some assembly artifacts affecting several contigs. For example, of three contigs containing more than 100 clones, the largest (Ctg148, 185 clones) most probably contains a large proportion of wrongly assembled clones. The sequence of several BAC-ends (BES) of clones from this contig revealed tandem repeat sequences of DNA in the form of ribosomal RNA genomic regions [data not shown]. Similar results have been obtained with other repetitive sequences, such as retrotransposons, analyzing BES from other problematic contigs.

### Anchorage of the BAC library to the genetic map

A total of 215 genetic markers (117 RFLPs, 96 SNPs and 2 SSRs, all mapping to a single locus except 10 that mapped on two separate linkage groups) were used to anchor 845 BAC clones from our genomic library to the genetic map [Table 3]. Genetic markers were selected from previous versions of the PI 161375 × T111 melon genetic map, mainly RFLPs [45] and SNPs [20,46,47]. Selected genetic markers were homogeneously distributed along the melon genetic map. A BAC pooling strategy and PCR-based library screening, using pairs of oligonucleotides designed for each marker, were used to identify positive BAC clones. A complete list of all markers analyzed together with their bibliographical references is in Additional file 1 Table S1. When used to screen all BAC superpools from the library, 37 pairs of primers (17% of all markers tested) failed to amplify, even though they produced amplification bands when tested against melon genomic DNA. This points to the existence of genomic regions poorly, or not represented in the BAC library used. A total of 178 markers (100 RFLPs, 76 SNPs and 2 SSRs) could be linked to 845 BAC clones, with 25 of these markers linked to a single clone while 153 markers linked to more than one clone. This gave a total of 820 BAC clones grouped in 153 sets of overlapping clones containing one common marker (from now on referred to as "PCR contigs", as opposed to "FPC contigs") [Table 3].

**Table 3:** Summary of the *C. melo *anchored genetic map.

													Markers								
**Linkage Group:**	**I**	**II**	**III**	**IV**	**V**	**VI**	**VII**	**VIII**	**IX**	**X**	**XI**	**XII**	**mapping**	**TOTAL**							

													**two LG**								
**RFLP**																					
**Total Analyzed**	8	14	10	10	7	10	6	11	8	8	10	5	10	117							
	
**Non-Anchored^a^**	1	2	2	3	1	1	1	2	2	0	2	0			17						
	
**Anchored to BACs**	7	12	8	7	6	9	5	9	6	8	8	5	10			100					
**Contigs**	7	11	7	6	6	8	5	7	5	7	7	5	10				91				
**Singletons**	0	1	1	1	0	1	0	2	1	1	1	0	0					9			
	
**Number of positive BAC clones**	45	68	38	28	47	52	32	43	37	33	37	22	56						538		
	
**Anchored to Physical Map (FPC):**																					
**To Contigs**	6	10	7	7	6	9	5	7	5	5	7	4	10							88	
**Only to Singletons**	1	1	0	0	0	0	0	1	0	2	1	1	0								7

**SNP**																					
**Total Analyzed**	9	5	7	9	5	11	12	9	10	6	9	4		96							
	
**Non-Anchored^a^**	1	0	0	1	3	4	2	2	1	1	3	2			20						
	
**Anchored to BACs**	8	5	7	8	2	7	10	7	9	5	6	2				76					
**Contigs**	7	5	5	6	1	3	9	6	7	5	5	2					61				
**Singletons**	1	0	2	2	1	4	1	1	2	0	1	0						15			
	
**Number of positive BAC clones**	34	23	24	29	5	19	46	24	33	23	30	14							304		
	
**Anchored to Physical Map (FPC):**																					
**To Contigs**	8	5	6	7	1	4	10	6	9	4	6	2								68	
**To Singletons**	0	0	0	0	1	1	0	1	0	1	0	0									4

**SSR**																					
**Total Analyzed**	0	0	0	0	0	2	0	0	0	0	0	0		2							
	
**Anchored to BACs**						2										2					
**Contigs**						1											1				
**Singletons**						1												1			
	
**Number of positive BAC clones**						3													3		
	
**Anchored to Physical Map (FPC):**																					
**To Contigs**						1														1	
**To Singletons**						1															1

													**TOTAL:**	215	37	178	153	25	845	157	12

^a ^Markers that failed to amplify when tested in the BAC library even though PCR amplification bands were detected when parental

genomic DNA was used as a positive control

**Figure 1:**
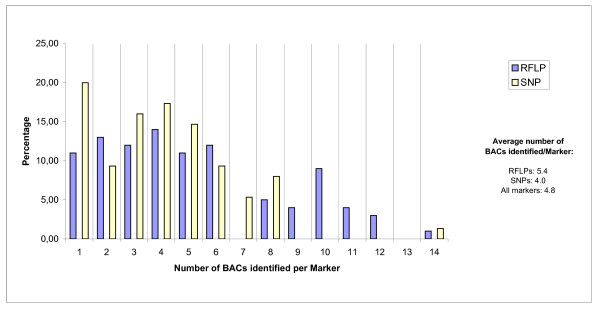
**Distribution of linked markers according to the number of anchored BACs per marker**.

The average number of BACs amplified per marker was 4.8. This is lower than expected based on the estimated genomic coverage of our library (5.7). This is a further indication of the absence of several genomic loci or overestimation of representation in the library used. A distribution of the number of positive BACs found per marker shows a two-zone distribution with about 79% of markers evenly distributed in the 1-6 BAC/marker range while the remaining markers are linked on average to 9-10 BAC/marker, probably reflecting the fact that about 20% of the primers used amplified duplicated or closely related genomic sequences [Figure 1]. In fact, while only 15% of the SNP markers were linked to contigs of more than six BAC clones, 26% of all anchored RFLPs were linked to between 7 and 14 clones. This indicates that the RFLP-derived primers were twice as prone to amplifying duplicated sequences as the SNP-derived primers. While an average of 5.4 clones were linked to RFLP markers, only 4.0 were linked to SNPs. Based on these results, it can be tentatively assumed that the genomic coverage of the library is somewhere in the 4-5 range.

### Integration of the physical and genetic maps

**Figure 2:**
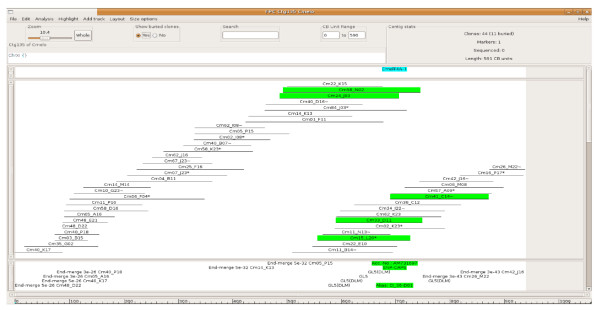
**FPC contig 135 anchored to the linkage group XI of the *C. melo *genetic map**. The contig consists of 44 clones spanning an estimated region of 800 kb of the *C. melo *genome. Clones highlighted in green are positive to the SNP marker CmelF4A-1 (linkage group XI) according to the information derived from the genetic map.

The anchorage of BAC clones to the genetic map was used to establish a link between the fingerprint-based physical map and the genetic linkage map. To this end, information regarding anchored genetic markers and their chromosome locations was introduced in the FPC database for all anchored BAC clones successfully fingerprinted. As a result, 169 genetic markers were anchored to the physical map, with 157 of them linked to FPC contigs of several lengths while 12 linked only to singletons [Tables 1 and 2]. Figure 2 shows an example of an FPC contig anchored to a melon linkage group using information derived from the genetic map. One hundred and fifty-eight of the anchored FPC contigs were linked to just one marker, 11 to two markers, and two contigs to three markers. All but 30 genetic markers mapped a single FPC contig or singleton: 25 markers mapped two separate contigs/singletons, 4 markers were each linked to three FPC contigs/singletons and one marker to four contigs. Therefore, 18% of all markers anchored to the physical map were linked to more than one FPC contig/singleton, a figure in accordance to the above estimation of the number of primers amplifiying duplicated or closely related genomic regions.

**Figure 3:**
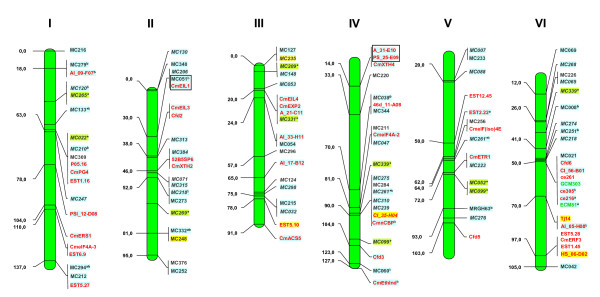
**A representation of the genetic map of the *C. melo *genome (PI161375 × "Piel de Sapo" [T111]) with markers anchored to the physical map**. RFLP markers are shown in black, SNPs in red, SSRs in green. *: RFLP markers mapping at two different map locations. Markers for which no hits were found in BACs are on white background; those anchored to BACs lacking the 5E/SNaPshot profile are shown on yellow background whilst those anchored to the FPC map appear on blue background; markers that map at two different map positions but have been anchored to a single FPC contig appear with green background at both map positions. Markers anchored to the same BAC contigs are in a black square. ^a^: markers anchored only to FPC singletons; ^b^: markers anchored to more than one FPC contig. Linkage groups are numbered according to the *C. melo *map of Deleu *et al*. [20]. Map distances are indicated on the left in cM. Markers in italics have been placed in an approximate position from Oliver *et al*. [45].

**Figure 4:**
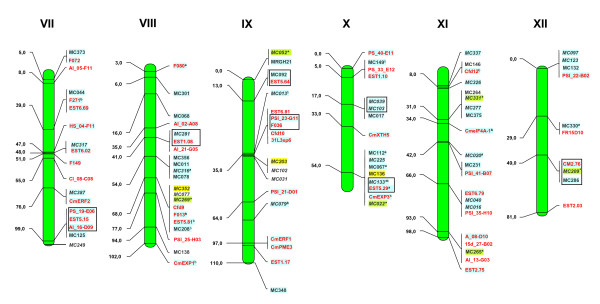
**A representation of the genetic map of the *C. melo *genome (PI161375 × "Piel de Sapo" [T111]) with markers anchored to the physical map**. RFLP markers are shown in black, SNPs in red, SSRs in green. *: RFLP markers mapping at two different map locations. Markers for which no hits were found in BACs are on white background; those anchored to BACs lacking the 5E/SNaPshot profile are shown on yellow background whilst those anchored to the FPC map appear on blue background; markers that map at two different map positions but have been anchored to a single FPC contig appear with green background at both map positions. Markers anchored to the same BAC contigs are in a black square. ^a^: markers anchored only to FPC singletons; ^b^: markers anchored to more than one FPC contig. Linkage groups are numbered according to the *C. melo *map of Deleu *et al*. [20]. Map distances are indicated on the left in cM. Markers in italics have been placed in an approximate position from Oliver *et al*. [45].

In all, 2,215 fingerprinted BAC clones, distributed in 183 contigs/singletons and representing 55 Mb or 12.2% of the melon genome, have been positioned in unique loci of the genetic map (except for seven contigs/singletons associated to markers that map to two separate chromosome locations, and so cannot be assigned to a single locus). On average, 175 BAC clones, 15 contigs and 4.4 Mb have been anchored to unique loci for every *C. melo *linkage group. All information regarding contig estimated length, contig clone number and chromosome location of all FPC contigs or singletons anchored to genetic markers can be found in Additional file 2 Table S2. A representation of the anchored genetic map together with information on which markers are linked to FPC contigs is shown in Figure 3&4, while more detailed information regarding linkage group distribution of the type and number of genetic markers analyzed and linked to both physical and anchored genetic maps is given in Table 3. The resulting genetic-anchored melon physical map FCP database is available for download at http://melonomics.upv.es/static/files/public/physical_map/.

### Validation of physical map contigs

The comparison between PCR and FPC contigs served as a quality control of the physical map building. Column 8 in Additional file 2 Table S2 shows the number of clones of every PCR contig anchored to any genetic marker while column 9 shows how many of those clones are also present in the FPC contigs anchored to the correspondent marker. Bearing in mind that an estimated 20% of all primer pairs tested produced positive BACs belonging to two or even three separate genomic regions and that, accordingly, 18% of all FPC-anchored markers are linked to more than one FPC contig or singleton, the degree of coincidence between PCR and FPC contigs has been computed using data from markers linked to a single FPC contig/singleton. As an average, 75% of those clones belonging to the same PCR contig are predicted to overlap according to the FPC information. Therefore, as 20% of the library clones were not successfully fingerprinted and so are absent from the physical map, we estimate that the degree of coincidence between PCR and FPC contigs is around 80%. However, when distinguishing duplicated or closely related genomic regions or gene families, the PCR screening procedure used to anchor the BAC library to the genetic map - i.e. PCR amplification of regions 100-1,000 bp long - should be much less efficient than fingerprinting whole BAC genomic regions. This can account for several of those clones that belong to PCR contigs but are absent from the correspondent FPC contig and, if so, the degree of coincidence between our physical and anchored genetic maps would be higher than the above estimation.

As another way to validate the physical map, the linkage group position of those markers linked to a common FPC contig was compared. As described above, of all FPC contigs/singletons linked to genetic markers, only 14 were anchored to more than one marker each. Of these, Ctg6, Ctg7, Ctg25, Ctg26, Ctg91, Ctg118, Ctg128, Ctg140 and singleton Cm19_G01 [Additional file 2 Table S2] are each linked to two or three markers that have the same position in the genetic map [Figure 3&4, markers in black-edged squares], which validates these FPC contigs as good assemblies. Two additional contigs, Ctg150 and Ctg147, were each linked to pairs of markers with conflicting genetic positions. An analysis of the construction of each contig revealed the merging of previous smaller contigs during the final steps of the autobuild process at greater cutoffs/lower stringency (1e-20 and 1e-15), which explains the presence of incompatible markers in these contigs. Another contig, Ctg898, is also linked to a set of two incompatible genetic markers. However, in this case, the clones belonging to the corresponding PCR contig coincide, and so they probably map two separate genomic regions that share some degree of sequence identity. The last two contigs, Ctg148 and Ctg 149, are also linked to conflicting sets of genetic markers (both markers linked to Ctg148 map to linkage group XI but at genetic distances much greater than the physical length of the contig), but this cannot be explained by low stringency automerge.

## Conclusions

Here we describe the physical map of *Cucumis melo*, the first example of a Cucurbitaceae physical map so far developed. The map FPC database is available for download at http://melonomics.upv.es/static/files/public/physical_map/. As melon is a species with an average sized genome (445 Mbp), the 5 enzyme/SNaPshot HICF method is the natural choice to build a physical map providing significant coverage of the genome. Using a BAC library representing about five genomic equivalents, the estimated physical length of the map is 407 Mb (0.91 × coverage of the melon genome). The anchorage of the BAC library to the available genetic map has allowed the genetic positioning of 183 physical contigs that represent an estimated 12% of the melon genome. The data presented is already helping to improve the quality of the available genomic sequence of this species, with considerable research effort to obtain a complete genomic sequence of the melon currently being carried out in Spain, adopting a whole-genome shotgun approach based on new generation massive sequencing data.

## Methods

### Source BAC library

A *Bam*HI BAC library from the double-haploid melon line 'PIT92' was previously developed in our laboratory [10]. With 23,040 BAC clones distributed in sixty 384-well plates, an average insert size of 139 kb and 20% empty clones, the library represents 5.7 genomic equivalents of the haploid melon genome.

### Choice of genetic markers for PCR anchoring

Two-hundred and fifteen genetic markers from the PI 161375 × T111 melon genetic map were selected for anchoring to the physical map. Markers were evenly distributed along the 12 linkage groups. One hundred and seventeen RFLPs, 96 SNPs and 2 SSRs, previously described [20,45-49], were selected (Additional file 1 Table S1).

### BAC library 3D pooling and PCR anchoring

The 60 library 384-well plates were replicated in 96-well plates, with 4 clones in each well and 200 μl 2 × LB medium. After overnight growth, a sample of 50 μl was taken from each well from one plate, added to 2.5 ml LB containing chloramphenicol at 12.5 μg/ml and grown overnight at 37°C. DNA minipreps of the bacterial culture was as described by [50], resulting in 60 DNA pools, each containing 384 BAC clones. DNA from 5 pools was combined to give 12 DNA superpools. The remaining bacterial culture in the 96-well plates was used for row and column DNA pools for each plate. Each row pool contained 48 BAC clones while each column pool contained 32 BAC clones.

A pair of specific primers was designed for each genetic marker and a first round of PCR, using 0.5 μl BAC miniprep, was performed with the 12 DNA superpools, followed by a second with the DNA pools from the positive DNA superpools, and a third with the 12 column pools and 8 row pools from each positive plate pool. The clone coordinates were those of clones in the reduced 60 × 96-well library. An additional round of PCR established the final coordinates in the original BAC library for each positive. Positive controls using genomic DNA from the PIT92 parental lines (PI161375 and T111 'Piel de Sapo') were included in each round of PCR. The final amplified bands were sequenced using one of the primers used for PCR amplification and the sequences compared to that of the genomic markers analyzed to confirm the positives.

### BAC DNA isolation and fingerprinting

Four μl of each BAC clone from a 384-well plate was inoculated into a 384-well plate containing 70 μl 2 × LB plus 12.5 μg/ml chloramphenicol. Plates were covered with adhesive gas permeable seals (Thermo Scientific) and incubated at 37°C, 250 rpm for 21-24 h. The following day, 15 μl of each BAC clone from the preinoculated 384-well plate were inoculated into four 96 deep-well plates, containing 1.2 ml LB plus chloramphenicol, and grown at 37°C, 300 rpm for 16 h. BAC DNA was obtained using a modified alkaline method followed by purification using the Microplate Unifilter+Uniplate Devices from Whatman Inc. DNA was resuspended in 30 μl of autoclaved milliQ water, with a typical final yield around 0.8-2 μg of DNA. Ten μl of the miniprep DNA was then digested using *Bam*HI, *Eco*RI, *Nde*I, *Xba*I and *Hae*III enzymes (New England Biolabs) for three hours at 37°C, and the mixture of restriction fragments labeled using the SNaPshot Multiplex kit (Applied Biosystems). The labeled fragments were precipitated by adding chilled 95% ethanol and resuspended in 10 μl of water prior to a final purification step using genClean plates (Genetix). A mixture of 9.95 μl HiDi™ (Applied Biosystems) and 0.05 μl 500 LIZ^® ^marker (Applied Biosystems) was added to each sample. The DNA was denatured by heating for 2 min at 95°C and then loaded in an ABI3730 DNA sequencer (Applied Biosystems) for fragment separation by capillary electrophoresis, using the Genescan LIZ500 marker as an internal standard for fragment sizing. The electrochromatograms were analyzed using GeneMapper 3.5 (Applied Biosystems).

### Fingerprint data collection and physical map construction

A text file containing area, height and size data was exported from GeneMapper and background, poor quality fingerprints, vector bands and empty clones removed using the FPB software [44]. The FPB parameters were as follows: minimum bands, 40; tolerance, 0.4; multiplying factor, 30; peak width, 15; band sizes from 50 to 500; size per color, from 5 to 250; color background, 50; fixed threshold, 500; first and last values, 3 and 7; low index: 60; color offset, 0, 15,000, 30,000 and 45,000.

The FPC processed data were then assembled using the FPC v9.3 Software [[25]; http://www.agcol.arizona.edu/software/fpc/]. Tolerance was set at 12 (= 0.4 × 30) and the contigs assembled with the Best Contig parameter set at 100. Several preliminary assemblies were performed in order to determine the optimal cut-off value, using cut-off values of 1e-35, 1e-45, 1e-55 and 1e-65, minimizing the number of contigs without significantly increasing the number of Q-clones. The building of the physical map was according to a standard iterated procedure [32,34]: initially at a sufficiently stringent cutoff to ensure the validity of the contigs, then gradually merging the contigs at successively greater cutoff values. Based on the results, the first automatic assembly was performed using a Sulston score of 1e-45. This was followed by breaking contigs containing more than 5% of Q-clones, applying the DQer function. The resulting contigs were end-merged using the 'End to End' function, setting the 'FromEnd' parameter to 50, 'Match' to 2 and the cutoff value to 1e-40. Finally, singletons were added to contig ends using the 'Singles to End' function. In several additional rounds of End to End/Singles to End the cutoff values were set to 1e-35, 1e-30, 1e-25, 1e-20 and 1e-15, with the DQer function applied when necessary after each round to break up those contigs containing more than 10% Q-clones.

## Authors' contributions

VMG conducted BAC library pooling and screening, DNA extractions and BAC fingerprinting, assembled the physical map and drafted the manuscript, JGM chose the genetic markers for PCR anchoring, participated in the project design, discussion of results and helped draft the manuscript, PA participated in the project design and discussion of results, PP conceived and coordinated the project, and helped draft the manuscript. All authors read and approved the final manuscript.

## Supplementary Material

Additional file 1 Table S1: List of genetic markers used in genetic map anchoring and physical map building. Click here for file

Additional file 2 Table S2: List of FPC contigs and singletons linked to genetic markers. Click here for file
